# Localization of NG2 immunoreactive neuroglia cells in the rat locus coeruleus and their plasticity in response to stress

**DOI:** 10.3389/fnana.2014.00031

**Published:** 2014-05-14

**Authors:** Mohsen Seifi, Nicole L. Corteen, Johannes J. van der Want, Friedrich Metzger, Jerome D. Swinny

**Affiliations:** ^1^Institute for Biomedical and Biomolecular Sciences, School of Pharmacy and Biomedical Sciences, University of PortsmouthPortsmouth, UK; ^2^Department of Cell Biology, University Medical Centre Groningen, University of GroningenGroningen, Netherlands; ^3^Electron Microscopy and Histology, Department of Laboratory Medicine, Children's and Women's Health, Norwegian University of Science and TechnologyTrondheim, Norway; ^4^Pharma Research and Early Development, DTA Neuroscience, F. Hoffmann-La Roche LtdBasel, Switzerland

**Keywords:** OPC, noradrenaline, brainstem, glia, GABAA receptor

## Abstract

The locus coeruleus (LC) nucleus modulates adaptive behavioral responses to stress and dysregulation of LC neuronal activity is implicated in stress-induced mental illnesses. The LC is composed primarily of noradrenergic neurons together with various glial populations. A neuroglia cell-type largely unexplored within the LC is the NG2 cell. NG2 cells serve primarily as oligodendrocyte precursor cells throughout the brain. However, some NG2 cells are in synaptic contact with neurons suggesting a role in information processing. The aim of this study was to neurochemically and anatomically characterize NG2 cells within the rat LC. Furthermore, since NG2 cells have been shown to proliferate in response to traumatic brain injury, we investigated whether such NG2 cells plasticity also occurs in response to emotive insults such as stress. Immunohistochemistry and confocal microscopy revealed that NG2 cells were enriched within the pontine region occupied by the LC. Close inspection revealed that a sub-population of NG2 cells were located within unique indentations of LC noradrenergic somata and were immunoreactive for the neuronal marker NeuN whilst NG2 cell processes formed close appositions with clusters immunoreactive for the inhibitory synaptic marker proteins gephyrin and the GABA-A receptor alpha3-subunit, on noradrenergic dendrites. In addition, LC NG2 cell processes were decorated with vesicular glutamate transporter 2 immunoreactive puncta. Finally, 10 days of repeated restraint stress significantly increased the density of NG2 cells within the LC. The study demonstrates that NG2 IR cells are integral components of the LC cellular network and they exhibit plasticity as a result of emotive challenges.

## INTRODUCTION

The brainstem locus coeruleus (LC)-noradrenergic system (Berridge and Waterhouse, 2003) is an integral orchestrator of the cognitive loop of the stress response which ensures optimal decision making in the face of adversity (Valentino and Van Bockstaele, 2008). This LC-noradrenergic stress response is generally an adaptive mechanism which allows the individual to contend with daily challenges and is thus essential for survival. However, chronic exposure to stressors is a risk factor for developing a range of mental health disorders such as anxiety and depression (Friedmann et al., 2006; Itoi and Sugimoto, 2010). The precise mechanisms underlying the adaptive or resilience responses to stressors compared to those which manifest in deleterious consequences remain elusive (Krystal and Neumeister, 2009; Russo et al., 2012).

A central molecule in the LC-noradrenergic stress pathways is the stress related hormone, corticotrophin releasing hormone (CRH; Valentino et al., 1992, 1993, 1998) which directly activates LC noradrenergic neurons (Valentino et al., 1983; Jedema and Grace, 2004; Swinny et al., 2010). It is currently unclear whether other cell-types within the LC nucleus, apart from the principal noradrenergic neurons, are responsive to stressors. The LC is composed of the principal noradrenergic neurons as well as neurochemically distinct non-noradrenergic neurons (Aston-Jones et al., 2004; Corteen et al., 2011). Apart from neurons, glia, in particular, astrocytes, have also been shown to be integral to coordinated LC function (Alvarez-Maubecin et al., 2000; Ballantyne et al., 2004). However, a neuroglia cell that is unexplored within the LC is the NG2 cell (Butt et al., 2002).

Nerve/glial antigen 2 (NG2) is a chondroitin sulfate proteoglycan predominantly expressed in the brain by a population of cells called NG2 cells (Raff et al., 1983; Stallcup and Beasley, 1987). NG2 cells are considered to be oligodendrocyte precursor cells (OPCs) since they express OPC markers (Nishiyama et al., 1996; Reynolds and Hardy, 1997), give rise to oligodendrocytes (Dimou et al., 2008; Rivers et al., 2008; Kang et al., 2010; Clarke et al., 2012) and thus are thought to be primarily involved in myelination. A population of glial cells with the characteristics of OPC persists into adulthood after most of the myelination within the CNS is complete. Adult NG2 cells are distributed in both gray and white matter regions of the brain (Nishiyama et al., 1999), are in synaptic contact with neurons (Bergles et al., 2000; Ge et al., 2006) and proliferate in response to brain injury (Levine et al., 2001). These data suggest that NG2 cells may participate in processes other than myelination and are capable of dynamically responding to their environment such as changing patterns of neighboring neuronal activity. An added layer of complexity is their purported multi-potency in terms of their ability to differentiate into cell-types other than oligodendrocytes such as neurons (Belachew et al., 2003; Aguirre and Gallo, 2004; Rivers et al., 2008; Guo et al., 2009), although this remains contentious see Kang et al. (2010) and Richardson et al. (2011). While the proliferative response of NG2 cells to traumatic brain injury is well documented, whether such dynamic properties exist following emotive insults such as psychosocial stress is largely unexplored. Since the LC is a central locus of the stress response, we explored this question using this nucleus. The expression of NG2 cells specifically within the LC is yet to be reported on. Therefore, the aim of the study was to first neurochemically and anatomically characterize NG2 cells within the cellular networks of this nucleus and then determine their response to repeated stress.

## MATERIALS AND METHODS

All procedures involving experimental animals were performed in accordance with the Animals (Scientific Procedures) Act, 1986 (UK) and associated procedures. Every effort was made to minimize any pain or discomfort to the animals.

### TISSUE PREPARATION

Male Wistar rats were used throughout the study. Anesthesia was induced with isoflurane and maintained with phenobarbitone (1.25 mg/kg of bodyweight; i.p.). The animals were perfused transcardially with 0.9% saline solution for 3 min, followed by 15 min fixation with a fixative consisting of 1% paraformaldehyde, 15% v/v saturated picric acid, in 0.1 M phosphate buffer (PB), pH 7.4. This fixation protocol allowed for the visualization of immunoreactivity for both NG2 and synaptically localized proteins. The brains were kept in the same fixative solution overnight at 4°C. An important aspect of the study was to determine the association between NG2 cell profiles and LC noradrenergic somata and dendrites. Since the LC noradrenergic dendrites project preferentially in the rostro-caudal plane (Shipley et al., 1996; Travagli et al., 1996), the LC was thus sectioned in the horizontal plane. A Vibratome was used to prepare 70 μm thick tissue sections which were then stored in 0.1 M PB containing 0.05% sodium azide.

### IMMUNOHISTOCHEMICAL REACTIONS

The non-specific binding of secondary antibodies was blocked by incubating sections with 20% normal horse serum, diluted in TRIS-buffered saline containing 0.3% Triton-X100 (TBS-Tx) for 2 h at room temperature. Tissue sections were incubated with a range of primary antibodies documented in **Table 1**. All antibodies were diluted in TBS-Tx, and incubated for 24 h at 4°C. After washing with TBS-Tx, sections were incubated in a mixture of appropriate secondary antibodies conjugated with either Alexa Fluor 488 (Invitrogen, Eugene, OR, USA), indocarbocyanine (Cy3; Jackson ImmunoResearch), and indodicarbocyanine (Cy5; Jackson ImmunoResearch) for 2 h at room temperature. Sections were then washed in TBS-Tx and mounted in Vectashield (Vector Laboratories, Burlingame, CA, USA). Method specificity was also tested by omitting the primary antibodies in the incubation sequence. To confirm the absence of cross reactivity between IgGs in double and triple immunolabeling experiments, some sections were processed through the same immunocytochemical sequence, except that only an individual primary antibody was applied with the full complement of secondary antibodies.

**Table 1:** Details of primary antibodies used in the study.

Antibody	Host	Dilution	Source	Specificity/reference
CRF	Guinea-pig	1:1000	Peninsula labs (T-5007)	Stanic et al. (2010), Armstrong et al. (2009)
Tyrosine hydroxylase	Sheep	1:3000	Abcam (AB113)	Raised to rat recombinant TH. Labeling pattern as published with other antibodies
NeuN	Mouse	1:1000	Millipore (MAP377)	Canola et al. (2007), Tippett et al. (2007)
NG2	Rabbit	1:1000	Millipore (AB5320)	Jiang et al. (2013), Palenski et al. (2013)
MBP	Rat	1:1000	Abcam (AB7349)	Yang et al. (2013), Kida et al. (2013)
NG2	Mouse	1:500	Millipore (MAB5384)	Sharma et al. (2012), Holopainen et al. (2012)
DBC	Goat	1:500	Santa cruz (SC-8066)	Marques-Torrejon et al. (2013), Bolos et al. (2013)
VGLUT2	Rabbit	1:2000	Synaptic systems (135403)	Affaticati et al. (2011), Zhou et al. (2007)
Gephyrin	Mouse	1:1000	Synaptic systems (147011)	Korber et al. (2012), Nair et al. (2013)
GABA-A alpha3 subunit	Rabbit	1:1000	Werner sieghart, antigen sequence a3N amino acids 1–11, R # 14/15, Bleed # 17/04/1997	Corteen et al. (2011)
GFAP	Mouse	1:500	Neuromab (75–240)	Xue et al. (2013)

### IMAGE ACQUISITION

Sections were examined with a confocal laser-scanning microscope (LSM710; Zeiss, Oberkochen, Germany) using either a Plan Apochromatic 63x DIC oil objective (NA1.4) or a Plan Apochromatic 100x DIC oil objective (NA1.46). Z-stacks were used for routine evaluation of the labeling. All images presented represent a single optical section. These images were acquired using sequential acquisition of the different channels to avoid cross-talk between fluorophores, with the pinholes adjusted to one airy unit for all channels. Images were processed with the software Zen2008 Light Edition (Zeiss, Oberkochen, Germany) and exported into Adobe Photoshop. Only brightness and contrast were adjusted for the whole frame, and no part of a frame was enhanced or modified in any way.

### REPEATED RESTRAINT STRESS

The LC plays a central role in mediating the stress response at the CNS level and modulating adaptive behavioral responses to future stressors (Valentino et al., 1983; Valentino and Van Bockstaele, 2008). In addition, LC dysregulation following exposure to severe, chronic stress is also implicated in maladaptive responses to future stressors and the development of mental illnesses such as anxiety and depression (Harro and Oreland, 2001; Berridge and Waterhouse, 2003). It is currently unclear whether the principal noradrenergic neurons within the LC are solely engaged in LC-stress responses or other cell-types, such as non-noradrenergic neurons or glia within the LC are implicated. We therefore investigated whether exposure to a mild stressor influenced the expression of NG2 immunoreactive profiles within the LC. A deliberately mild stress protocol was used which has been shown not to induce any anxiogenic behavior, thus suggestive of an adaptive response to future stressors (Dhabhar et al., 1997), with a view to associating NG2 cell plasticity with such cellular mechanisms. We therefore used a repeated, variable restraint stress protocol (Buynitsky and Mostofsky, 2009) since restraint stress has been shown to robustly engage not only the peripheral but also central stress pathways by increasing the expression of proteins mediating the stress response (Inoue et al., 1993). A total of 12 (six control and six stress) male rats, aged PND 60 were used in this part of the study. Stress animals were exposed to a variable restraint protocol in order to prevent habituation. On the first 2 days, the animals were placed in a rodent Plexiglas restrainer (Harvard Apparatus) for 30 min. On the third day, the animal was placed in the restrainer without securing the fastener, thus allowing the animal to escape with a certain amount of effort. Once emerged from the Plexiglas restrainer, the animal was left in the cage with the restrainer for the remainder of the 30 min. On the fourth day, the animal was placed in the test cage for 30 min together with the restrainer, but not restrained. This sequence was then repeated for 10 days. One day after the last stressor, the animals were assessed for measures of anxiety and locomotor activity using the elevated plus maze (EPM) according to standard protocols (Walf and Frye, 2007). The time spent in the open and closed arms, as well as the number of entries into each arm was quantified within a 5 min exposure to the EPM. Differences between the mean time spent in each arm and the number of entries into each arm between control and stress cohorts were assessed for statistical significance using the Mann-Whitney test. The animals were then prepared for histological analyses as below.

### QUANTIFICATION OF THE DENSITY OF NG2 CELLS WITHIN THE LC DURING DEVELOPMENT AND AS A RESPONSE TO STRESS

A total of six rats, three aged postnatal day (PND) 3 and three aged PND 60 were used to estimate the density of NG2 cells within the LC during postnatal development. Tissue from five control and five stress animals was used to quantify the effect of stress on the density of NG2 cells within the LC. Quantification of NG2 cell density was performed according to previously published methods (Corteen et al., 2011). Briefly, serial, horizontal sections of entire LC nuclei were prepared (70 μm-thick sections) using a Vibratome. In our pilot experiments we found no differences in the fluorescence intensity of NG2 immunoreactivity throughout the rostro-caudal and dorso-ventral extent of the LC nucleus. Therefore, 4 tissue sections per animal were used for counting NG2 cell numbers within the LC. TH-immunoreactivity was used to delineate the LC. Only those NG2 cells located within the nuclear core region of the LC were counted. At the magnification used, several fields of view (FOV) were required to image the full area of the LC nuclear core. For a FOV, confocal Z-stacks of TH, NG2 and NeuN immunoreactivity were acquired from the top to the bottom surfaces of the tissue section using a Plan Apochromatic 40X DIC oil objective (NA1.3) with each optical section within the Z-stack measuring 236 μm × 236 μm × 5 μm (*X*, *Y*, *Z*). All NG2-immunopositive cells within an optical section, as well as whether they expressed NeuN or were located within TH-immunopositive somatic indentations, were manually counted using ImageJ software (NIH). The numbers of NG2 cells for each optical section within a field of view were combined and the mean density ± SD for all FOV within and between sections were compared for statistical differences using Kruskal–Wallis one-way analysis of variance. These values were then pooled since there were no statistical differences between FOV, between tissue sections and animals of the same age. The density analysis is presented as the number of cells per 100,000 μm^2^ and the *N* values refer to the number of animals as a function of either age or exposure to stress. The differences of the mean NG2 cell densities between animals aged PND3 and 60 or between control and stress treatment was assessed for statistical significance using the unpaired Student’s *t-*test.

### QUANTIFICATION OF THE RELATIVE PROPORTION OF VGLUT2-, GEPHYRIN- AND GABA_A_R ALPHA3 SUBUNIT IMMUNOREACTIVE CLUSTERS WHICH ARE LOCATED IN APPOSITION TO NG2 IMMUNOREACTIVE PROFILES WITHIN THE LC

The quantitative method used is according to (Corteen et al., 2011) using *N* = 4 animals and, three sections per animal. The following immunohistochemical reactions were performed: (1) TH- NG2-VGLUT2; (2) TH-NG2-gephyrin; (3) TH-NG2- GABA_A_R alpha3 subunit. Three FOV were randomly selected within the LC nuclear core region of each tissue section. A Z-stack consisting of three optical sections was acquired for each FOV with a Plan Apochromatic ×100 (NA1.4) DIC oil immersion objective. The dimensions of the optical sections were 84.94 μm × 84.94 μm in the *X* and *Y* planes and 1 μm thick in the *Z* plane. Optical sections were spaced 5 μm apart in the *Z* plane. In all cases, triple immunofluorescence was acquired using sequential acquisition of the different channels. The number of clusters within an optical section, either alone or in contact with NG2 cell profiles was manually counted using ImageJ software or expressed as the number of clusters per 10,000 μm^2^.

## RESULTS

In the current study, we investigated the expression of NG2-expressing cells within the LC with a view to determining the anatomical relationships between such neuroglia cells and the principal noradrenergic neurons of this nucleus during development and following exposure to repeated stress.

### NG2 CELLS ARE LOCATED IN CLOSE PROXIMITY TO LC NORADRENERGIC SOMATA AND DENDRITES

**FIGURE 1:**
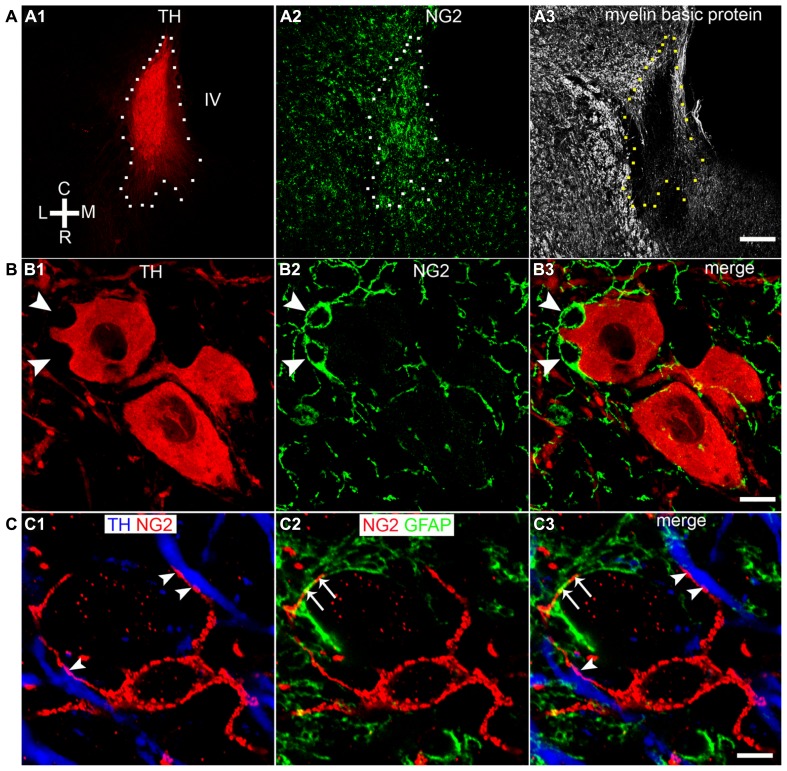
**NG2 cells are closely associated with the noradrenergic cells of the LC. (A1)** Overview of the LC in the horizontal plane visualized using tyrosine hydroxylase (TH) immunoreactivity (IR). **(A2)** NG2 IR within this region of the pons is enriched in the region occupied by the LC. **(A3)** IR for myelin basic protein (MBP) is distinctly lacking within the region occupied by the LC in line with evidence that LC noradrenergic neurons are un-myelinated. **(B1)** Shows a magnified view of a TH IR neuron which has a highly irregular shaped soma (arrowheads). **(B2)** Shows NG2 IR identifying small cellular profiles within the LC similar to NG2 cells described in other brain regions. **(B3)** Shows that the somata of these NG2 cells are positioned within indentations of the soma of the TH IR neuron. **(C1)** Shows an NG2 cells with its processes contacting TH immunopositive dendrites (arrowheads). **(C2)** Shows that the processes of the NG2 cell are also closely apposed to GFAP immunoreactive profiles (arrows) which are likely to be of astrocytic origin. **(C3)** is an overlay of **(C1)** and **(C2)** and suggests that NG2 cells are in contact with both neuronal and glial cell-types within the LC. Scale bars: **(A)** 200 μm; **(B)** 10 μm; **(C)** 5 μm. C, caudal; L, lateral; M, medial; R, rostral; IV, fourth ventricle.

Our initial investigations focused on the arrangement or location of NG2 cells in relation to the principal noradrenergic neurons of the LC (**Figure 1A1**). At low magnification, in adult tissue, visualization of pontine NG2 immunoreactivity revealed an enrichment of the signal within the region occupied by the LC, identified by tyrosine hydroxylase immunoreactivity, compared to neighboring brainstem nuclei, such as the mesencephalic trigeminal nucleus and Barrington’s nucleus (**Figure 1A2**). NG2 cells in adulthood are purported to serve as reservoir of OPCs and thus are primarily involved in myelination (Kang et al., 2010). However, LC noradrenergic neurons are thought to be un-myelinated (Aston-Jones et al., 1980; Olschowka et al., 1981). In addition, this study demonstrated a poor overlap of immunoreactivity for NG2 with myelin basic protein (MBP; **Figure 1A3**). Indeed, the region of the LC which expressed the highest levels of NG2 immunoreactivity, namely the nuclear core, showed only sparse labeling for MBP (**Figure 1A3**), which raises the question of the potential role of NG2 cells within this nucleus. NG2 immunoreactive cells had relatively small somata from which highly ramified processes emanated (**Figure 1B2**). A striking arrangement within the nuclear core of the LC was the location of a sub-population of NG2 cells within indentations (**Figure 1B1**) of tyrosine hydroxylase (TH) immunopositive somata suggesting a highly intimate relationship between LC noradrenergic neurons and a sub-population of NG2 cells (**Figure 1B3**). A further population of NG2 immunopositive cells which were not located in somatic indentations were randomly scattered throughout the extent of the LC with their processes located in close apposition to either TH immunopositive profiles or profiles immunopositive for glial fibrillary acidic protein (GFAP), a marker of astrocytes (**Figure 1C**). Collectively, these localization data suggest that NG2 cells are positioned to interact with the various cell-types contained within the LC nucleus.

### A SUB-POPULATION OF LC NG2 CELLS EXPRESSES NEURONAL MARKERS

**FIGURE 2:**
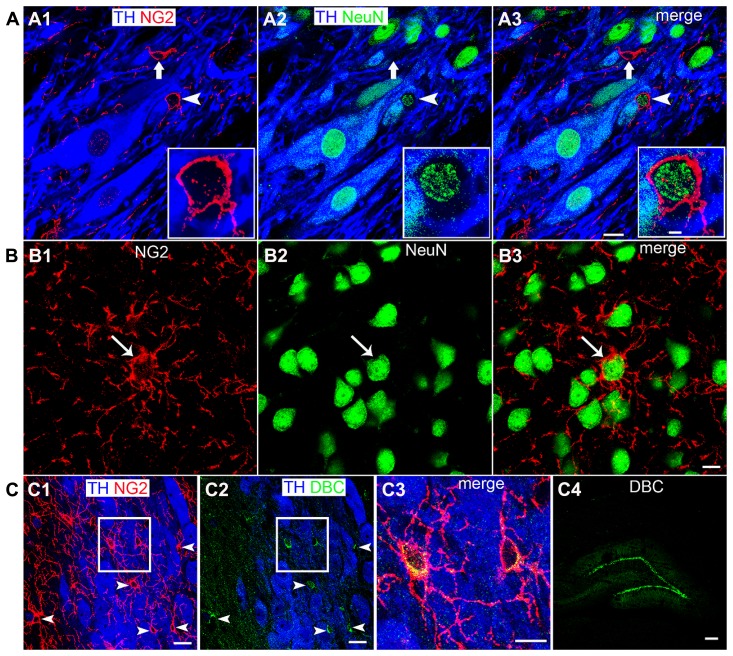
**A sub-population of LC NG2 cells expresses neuronal marker proteins. (A1)** NG2 IR is evident in a cell closely apposed to a TH IR soma (arrowhead) as well as an NG2 cell which is located in proximity to TH IR dendrites (arrow). **(A2)** Shows IR for the marker of mature neurons, NeuN, is localized to nuclei of TH IR neurons, non-TH IR neurons and the NG2 cell highlighted by the arrowhead. **(A3)** is an overlay of **(A1,2)** confirming that some NG2 cells (arrowhead) express the neuronal marker NeuN whereas others do not (arrow).The inserts are magnified regions of the NG2 cell highlighted by the arrowhead. **(B1)** shows NG2 expression and **(B2)** NeuN expression in a region of the piriform cortex. The NG2 IR is enriched on a cell body (arrow) and numerous radiating processes. **(B3)** shows that the isolated NG2 cell expresses the neuronal marker NeuN in agreement with (Rivers et al., 2008). **(C)** Shows that LC NG2 cells express doublecortin (DBC), a marker of migrating immature neurons. **(C1)** shows a number of NG2 cells (arrowheads) within the LC. **(C2)** shows that cells expressing DBC IR scattered throughout the LC (arrowheads) are those which exhibit NG2 IR in **(C1)**. **(C3)** is a magnified, merged view of the boxed region in **(C1)** and **(C2)** confirming that LC NG2 cells express DBC. **(C4)** shows the characteristic expression pattern of DBC in the dentate gyrus of the hippocampus which is thought to demonstrate migrating, newly born neurons in the adult brain. Scale bars **(A)** 10 μm, insert 2 μm; **(B)** 10 μm; (C1,2) 20 μm; **(C3)** 10 μm; **(C4)** 200 μm.

**FIGURE 3:**
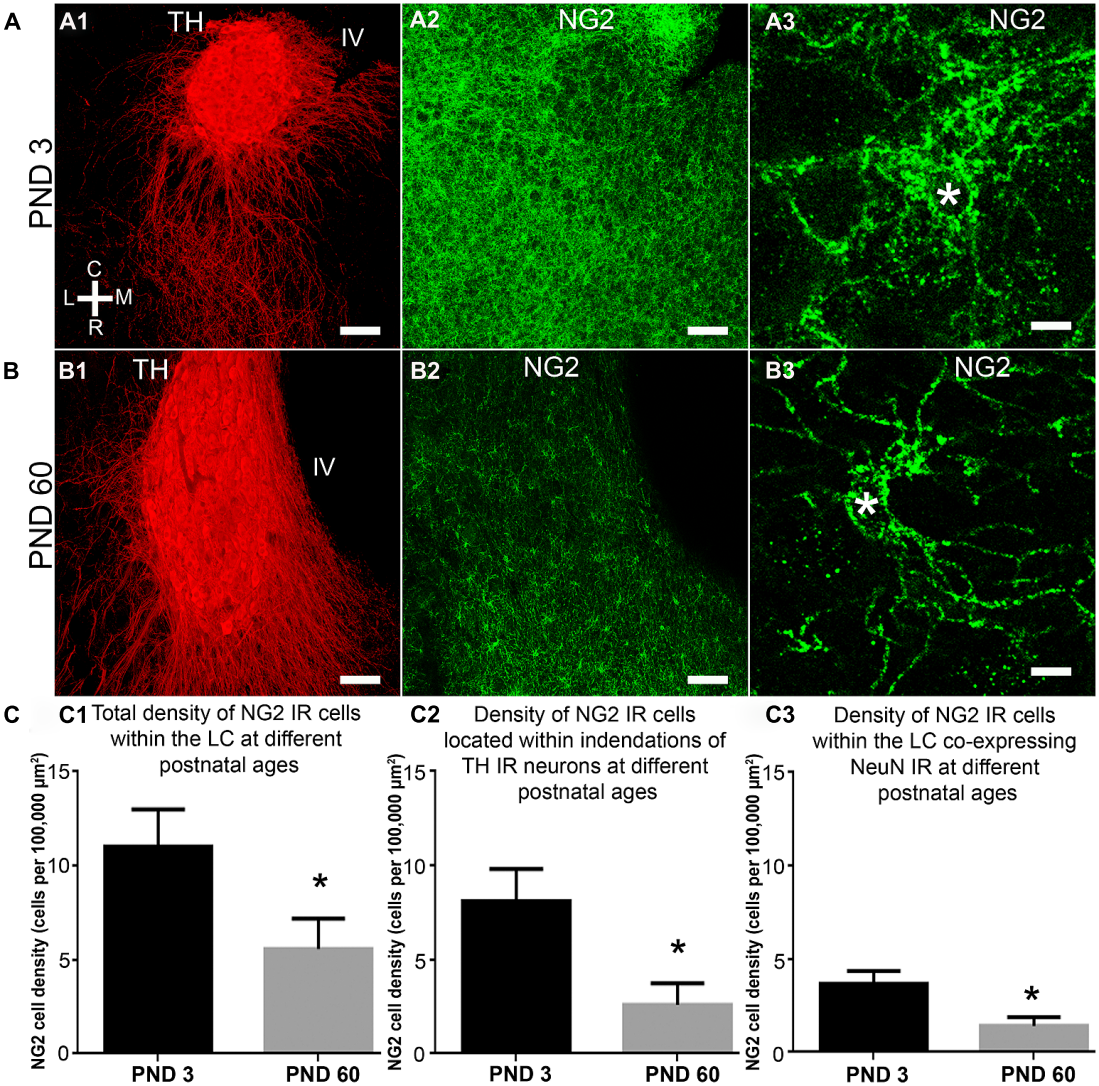
**NG2 cell density within the LC as a function of postnatal development, their location within somatic indentations and NeuN expression. (A1–3)** shows the level of NG2 IR in the LC and surrounding pons at postnatal day (PND) 3. Note the dense meshwork of NG2 cell processes in the magnified region shown in **(A3)**. **(B)** shows that at PND 60, NG2 IR is greatly reduced. The magnified NG2 cell in **(B3)** shows clearly defined, individual processes radiating from its cell body in contrast to the NG2 cells at PND 3 **(A3)**. Note that the tissue at different ages was reacted and imaged under identical conditions. This suggests an age-related remodeling of the morphology of NG2 cells. **(C1)** graphical representation of the density of NG2 cells within the LC at PNDs 3 and 60. Bars represent means and lines SEM. *P < 0.05, unpaired Student’s *t-*test; *N* = 3 animals. **(C2)** graphical representation of the density of NG2 cells located within indentations of LC noradrenergic somata at PNDs 3 and 60. Bars represent means and lines SEM. *P < 0.05, unpaired Student’s *t-*test; *N* = 3 animals. **(C3)** graphical representation of the density of NG2 cells expressing NeuN immunoreactivity at PNDs 3 and 60. Bars represent means and lines SEM. *P < 0.05, unpaired Student’s *t-*test; *N* = 3 animals. Scale bars **(A1–2,B1–2)** 60 μm; **(A3,B3)** 5 μm.

In cortical brain regions, heterogeneous populations of NG2 cells are evident based on not only their neurochemistry or progeny (Trotter et al., 2010) but also their functional characteristics, in particular, their capability of generating electrical activity reminiscent of neurons (Belachew et al., 2003; Karadottir et al., 2008). We used a range of neurochemical markers to investigate the molecular profiles of NG2 cells located within the LC. A sub-population of NG2 cells within the LC expressed the neuronal marker NeuN (**Figure 2A**; see **Figure 3** for quantification). Notably, all NeuN immunopositive NG2 cells were located within indentations of TH immunopositive somata although not all those NG2 cells which were located within indentations of TH immunopositive somata were NeuN immunopositive (see **Figure 3** for quantification). Under our experimental conditions, we did not detect any NG2 cells expressing NeuN in other cortical brain regions apart from isolated cells within the piriform cortex, in agreement with (Rivers et al., 2008; **Figure 2B**). NG2 cells within the LC were also immunopositive for doublecortin, a marker of migratory neuronal progenitors (**Figure 2C**). Such NG2-doublecortin immunopositive cells have been previously described in cortical brain regions (Tamura et al., 2007; Guo et al., 2010). Collectively, these data suggest that a select population of NG2 cells located within indentations of noradrenergic neurons express the molecular phenotypes of neurons.

### QUANTIFICATION OF LC NG2 CELL DENSITY AS A FUNCTION OF DEVELOPMENT, THEIR ASSOCIATION WITH NORADRENERGIC SOMATIC INDENTATIONS AND NEUN IMMUNOREACTIVITY

The LC undergoes extensive functional and morphological plasticity during postnatal development (Nakamura et al., 1987; Bezin et al., 1994). Furthermore, NG2 cell numbers in various brain regions have been shown to vary dynamically during brain maturation (Dawson et al., 2003). To determine whether NG2 cell numbers within the LC change during postnatal development, we examined NG2 immunoreactivity in the LC of animals aged PND 3 and 60 and quantified the mean NG2 cell density at these ages. Qualitatively, the level of LC NG2 immunoreactivity was strikingly more intense in tissue of animals aged PND 3 compared to that of PND 60, when the respective tissue sections were reacted and imaged under identical conditions (**Figures 3A,B**). Quantification of the density of NG2 cells located within the LC revealed a significant decrease during the postnatal period (mean ± SEM; PND 3, 11.4 ± 2 NG2 cell per 100,000 μm^2^ versus PND 60, 5.6 ± 0.4 NG2 cell per 100,000 μm^2^ (*N* = 4 animals for each age; *P* < 0.0001, unpaired Student’s *t-*test; **Figure 3C1**). This equates to a ~50% decrease in NG2 cells density during postnatal development. The density of those NG2 cells located within indentations of TH-immunopositive somata also decreased significantly during development (mean ± SEM; PND 3, 8.1 ± 1.7 NG2 cell per 100,000 μm^2^ versus PND 60, 2.5 ± 0.3 NG2 cell per 100,000 μm^2^, *P* < 0.0001, unpaired Student’s *t-*test; **Figure 3C2**). Thus, approximately 75% of LC NG2 cells were located within indentations of TH-immunopositive somata at PND 3 compared to only 45% at PND 60. Finally, the density of NeuN immunopositive NG2 cells also decreased postnatally (mean ± SEM; PND 3, 3.7 ± 0.7 NG2 cell per 100,000 μm^2^ versus PND 60, 1.4 ± 0.1 NG2 cell per 100,000 μm^2^, *P* < 0.0001, unpaired Student’s *t*-test; **Figure 3C3**). This equates to 34 versus 25% of NG2 cells expressing NeuN immunoreactivity at PND 3 and 60 respectively.

### LC NG2 CELL PROCESSES ARE CLOSELY ASSOCIATED WITH EXCITATORY AND INHIBITORY SYNAPTIC MARKER PROTEINS

**FIGURE 4:**
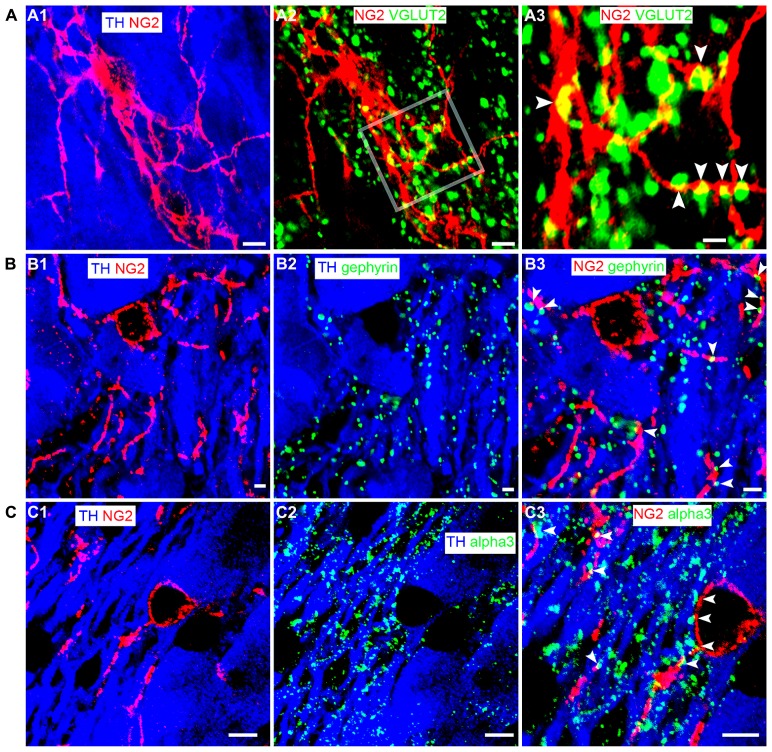
**LC NG2 cell processes are associated with excitatory and inhibitory synaptic proteins. (A1)** shows an NG2 cell and its processes together with TH IR cell bodies and dendrites. **(A2)** shows IR for the glutamatergic presynaptic protein, vesicular glutamate transporter 2 (VGLUT2) in relation to an NG2 cell. In **(A3)**, numerous VGLUT2 puncta (arrowheads) contact NG2 IR processes suggesting significant glutamatergic synaptic input onto NG2 cells in the LC. **(B1)** shows an NG2 cell and its processes closely trailing TH IR dendrites. **(B2)** shows the expression of gephyrin, a protein which anchors receptors (glycine and GABA-A) in inhibitory synapses. Gephyrin IR puncta are almost exclusively on TH IR dendrites. **(B3)** shows that numerous NG2 IR processes are apposed to gephyrin IR puncta (arrowheads) expressed on TH IR dendrites. **(C1)** shows an NG2 cell and its processes together with TH IR cell bodies and dendrites. **(C2)** shows IR for the alpha3 subunit, the major GABA-A receptor subunit expressed in the LC. Alpha3 subunit IR puncta are also exclusively on TH IR dendrites. **(C3)** shows that numerous NG2 IR processes appear to be apposed to alpha3 subunit IR puncta (arrowheads) expressed on TH IR dendrites. This suggests that NG2 cells target their processes to inhibitory synapses on noradrenergic neurons of the LC. Scale bars **(A1–2)** 5 μm, 1 μm; **(B1–3)** 2 μm; **(C1–3)** 5 μm.

NG2 cells have been shown to receive synaptic input from neurons (Bergles et al., 2000; Ge et al., 2006) suggesting communication between such cells and neighboring neurons. To gain a perspective, at the light microscopical level, on the potential mechanisms by which NG2 cells might communicate with neighboring cells within the LC, we used a range of synaptic marker proteins to examine the proximity of NG2 cell processes in relation to excitatory and inhibitory synapses within the LC. Clusters immunoreactive for the vesicular glutamate transporter 2 (VGLUT2), a protein expressed selectively in glutamatergic axon terminals, decorated NG2 cell processes (**Figure 4A**). We found only sparse evidence of vesicular GABA transporter (VGAT) immunoreactive clusters apposed to NG2 cell profiles. NG2 cell processes were closely apposed to clusters immunoreactive for gephyrin (**Figure 4B**), a protein which functions primarily to anchor glycinergic and GABAergic receptors at inhibitory synapses with its expression thus predictive of the location of such synapses (Tyagarajan and Fritschy, 2014). In addition, NG2 cell processes were also closely apposed to puncta immunopositive for the GABA-A receptor alpha3 subunit (alpha3-GABA_A_R; **Figure 4C**), the principal GABA_A_R subunit in noradrenergic neurons of the LC (Corteen et al., 2011). We found no convincing evidence of gap-junction expression between NG2 and TH-immunopositive profiles. While ultrastructural evidence using transmission electron microscopy is imperative for unequivocal confirmation, the location of NG2 cell processes in close proximity to clusters immunoreactive for synaptic proteins suggests a degree of synaptic input onto NG2 cells and synaptic contact with LC noradrenergic neurons.

**FIGURE 5:**
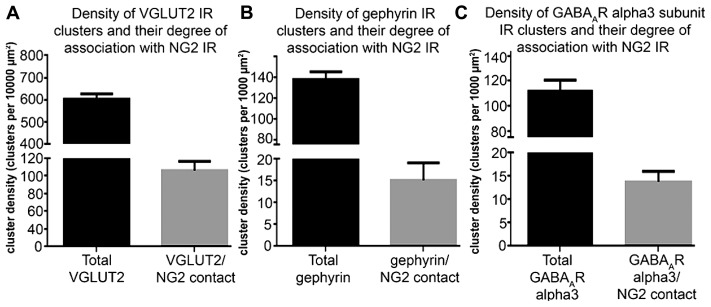
**Quantification of the density of contacts between VGLUT2, gephyrin and GABA_A_R alpha3 subunit immunoreactive clusters with NG2 cell profiles. (A)** Graphical representation of the density of total VGLUT2 immunoreactive clusters as well as the density of those VGLUT2 immunoreactive clusters which contact NG2 cell processes within the LC at PND 60. Bars represent means and lines SEM; *N* = 4 animals. **(B)** Graphical representation of the density of total gephyrin immunoreactive clusters as well as the density of those gephyrin immunoreactive clusters which contact NG2 cell processes within the LC at PND 60. Bars represent means and lines SEM; *N* = 4 animals. **(C)** graphical representation of the density of total GABA_A_R alpha3 subunit immunoreactive clusters as well as the density of those GABA_A_R alpha3 subunit immunoreactive clusters which contact NG2 cell processes within the LC at PND 60. Bars represent means and lines SEM; *N* = 4 animals.

To investigate the comparative association of these excitatory and inhibitory synaptic proteins with NG2 cell and noradrenergic profiles at the light microscopical level, we quantified the density of the contacts between the respective immunoreactivity profiles (**Figure 5**). The mean ± SEM density of VGLUT2 immunoreactive clusters within the nuclear core of the LC was 603 ± 11 clusters per 10,000 μm^2^ whereas the density of VGLUT2 immunoreactive clusters which contacted NG2 cell processes was 86 ± 5 clusters per 10,000 μm^2^ (**Figure 5A**) which suggests that approximately 14% of VGLUT2-containing glutamatergic axon terminals within the LC contact NG2 cells. The mean ± SEM density of gephyrin immunoreactive clusters within the nuclear core of the LC was 138 ± 3 clusters per 1000 μm^2^ whereas the density of gephyrin immunoreactive clusters which contacted NG2 cell processes was 15 ± 2 clusters per 1000 μm^2^ which equates to approximately 9% of total gephyrin immunoreactive clusters (**Figure 5B**). The mean ± SEM density of alpha3-GABA_A_R immunoreactive clusters within the nuclear core of the LC was 112 ± 4 clusters per 1000 μm^2^ whereas the density of alpha3-GABA_A_R immunoreactive clusters which contacted NG2 cell processes was 13 ± 1 clusters per 1000 μm^2^ which equates to approximately 13% of total alpha3-GABA_A_R immunoreactive clusters (**Figure 5C**).

### REPEATED RESTRAINT STRESS INCREASES NG2 CELL DENSITY IN THE LC

**FIGURE 6:**
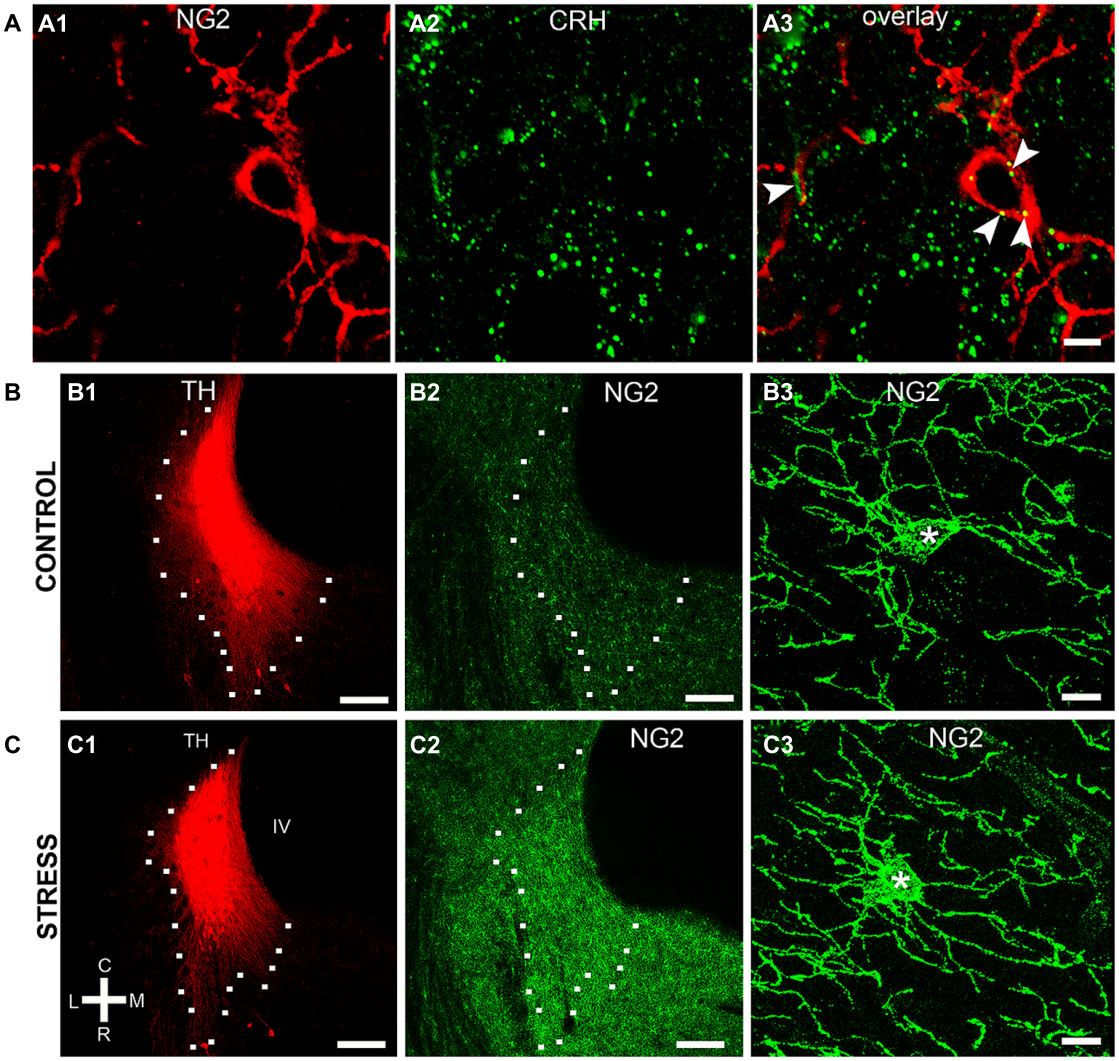
**Repeated restraint stress alters NG2 immunoreactivity within the LC. (A)** shows IR for the stress protein corticotrophin releasing hormone (CRH) in close apposition (arrowheads) to an NG2 cell. **(B1–2)** and **(C1–2)** show representative images of the levels of NG2 IR in the LC of control animals and animals exposed to 10 days of repeated restraint stress, respectively. Note the selective increase in the intensity of NG2 IR in the nuclear core and dendritic regions of the LC from animals exposed to stress. **(B3)** and **(C3)** are representative images of individual NG2 cells from control animals and animals exposed to stress. NG2 cells in animals exposed to stress appeared to exhibit a greater proportion of processes emerging from the cell body suggesting a stress-induced remodeling of NG2 morphology in addition to proliferation. Scale bars **(A)** 5 μm; **(B,C1,2)** 200 μm; **(B3,C3)** 10 μm. Asterisks denotes the cell body.

**FIGURE 7:**
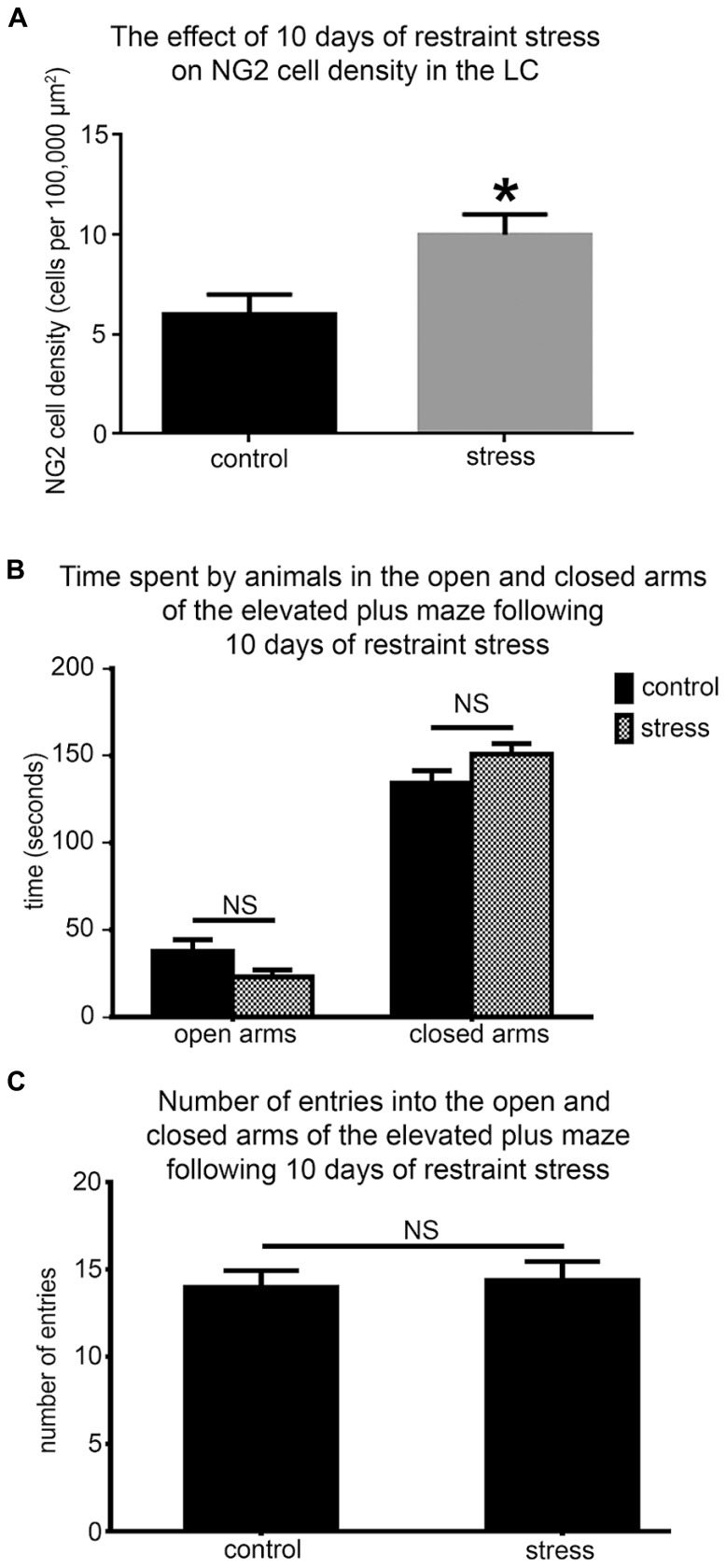
**Repeated restraint stress increases the density of NG2 cells within the LC but does not induce an anxiogenic or hyper-locomotor behavioral phenotype. (A)** Graphical representation of the density of NG2 cells within the LC of control and stress animals. Bars represent means and lines SEM. *P < 0.05, unpaired Student’s *t-*test; *N* = 5 animals. **(B)** Graphical representation of the times spent in the open (*P* = 0.43; unpaired Student’s *t-*test) and closed arms (*P* = 0.49; unpaired Student’s *t-*test) of the EPM in a 5 min trial by control and stress animals. Bars represent means and lines SEM. *N* = 6 animals. **(C)** Graphical representation of the total number of entries made into all the arms by control and stress animals. Bars represent means and lines SEM. *P* = 0.783, unpaired Student’s *t-*test; *N* = 6 animals.

NG2 cell density within the brain is highly dynamic since such cells continue to proliferate throughout adulthood (Dawson et al., 2003) with such proliferation accelerated in response to physical brain trauma (Levine et al., 2001). However, it is currently unclear whether NG2 cell density fluctuates in response to emotional trauma such as that which is known to contribute to mental illnesses, for example, environmental stress. If so, such NG2 cells plasticity is likely to occur in brain regions principally involved in the processing of stressful stimuli, such as the LC. The stress hormone, CRH directly innervates LC noradrenergic neurons (Valentino et al., 1992) and is thus the central mediator of the LC-stress system (Valentino et al., 1993). Surprisingly, we found CRF-immunopositive varicosities closely apposed to NG2 cells profiles (**Figure 6A**). Exposure of animals to 10 days of repeated restraint stress (30 min per day) resulted in a noticeable increase in the level of LC NG2 immunoreactivity in tissue from stress animals compared to tissue from controls, reacted and imaged under identical conditions (**Figures 6B,C**). In order to determine the reason for the stress-induced increase in LC NG2 cell immunoreactivity, we quantified the density of NG2 cells within the LC of control and stress animals. The density of LC NG2 cells was significantly higher in tissue from stress animals compared to control (mean ± SEM; control, 5.3 ± 0.4 NG2 cells per 100,000 μm^2^ versus stress, 10.8 ± 2 NG2 cells per 100,000 μm^2^, *N* = 5 animals; *P* < 0.05, unpaired Student’s *t-*test; **Figure 7A**). In addition, stress significantly increased the density of the subpopulation of NG2 cells which were located within indentations of TH immunopositive somata (mean ± SEM; control, 2.6 ± 0.3 NG2 cells per 100,000 μm^2^ versus stress, 3.7 ± 0.2 NG2 cells per 100,000 μm^2^, *N* = 5 animals; *P* = 0.0178, unpaired Student’s *t-*test, *N* = 5 animals in each group). However, the density of the subpopulation of NG2 cells which were also NeuN immunopositive was significantly less in tissue from stress animals compared to control (mean ± SEM; control, 1.7 ± 0.2 NG2 cells per 100,000 μm^2^ versus stress, 1.1 ± 0.1 NG2 cells per 100,000 μm^2^, *N* = 5 animals; *P* = 0.0185, unpaired Student’s *t-*test, *N* = 5 animals in each group). Finally, there did not appear to be any difference in the proportion of NG2 cells which were also immunopositive for doublecortin in tissue from control and stress animals (mean ± SEM; control, 5.1 ± 0.5 NG2 cells per 100,000 μm^2^ of which, 4.1 ± 0.5 were immunopositive for doublecortin, that is ~80% versus stress, 10.6 ± 0.8 NG2 cells per 100,000 μm^2^ of which, 8.6 ± 0.7 were immunopositive for doublecortin, that is ~ 81%.

In the context of this stress-induced increase in LC NG2 cell density, we next explored whether the baseline behavior of the animals was altered using the elevated plus maze to assess their levels of anxiogenic behavior and locomotor activity. There were no significant differences between the times spent in both the open (mean ± SEM; control, 30 ± 16 s versus 23 ± 7 sec; *P* = 0.42, *N* = 6 animals; unpaired Student’s *t-*test) and closed arms (mean ± SEM; control, 134 ± 18 s versus stress, 151 ± 14; *P* = 0.465, *N* = 6 animals; unpaired Student’s *t-*test; **Figure 7B**) as well as the total number of entries into each arms (mean ± SEM; control, 14 ± 1 entries versus stress 14 ± 1 entries; *P* = 0.42, *N* = 6 animals; unpaired Student’s *t-*test) of the elevated plus maze by control and stress animals (**Figure 7C**).

**FIGURE 8:**
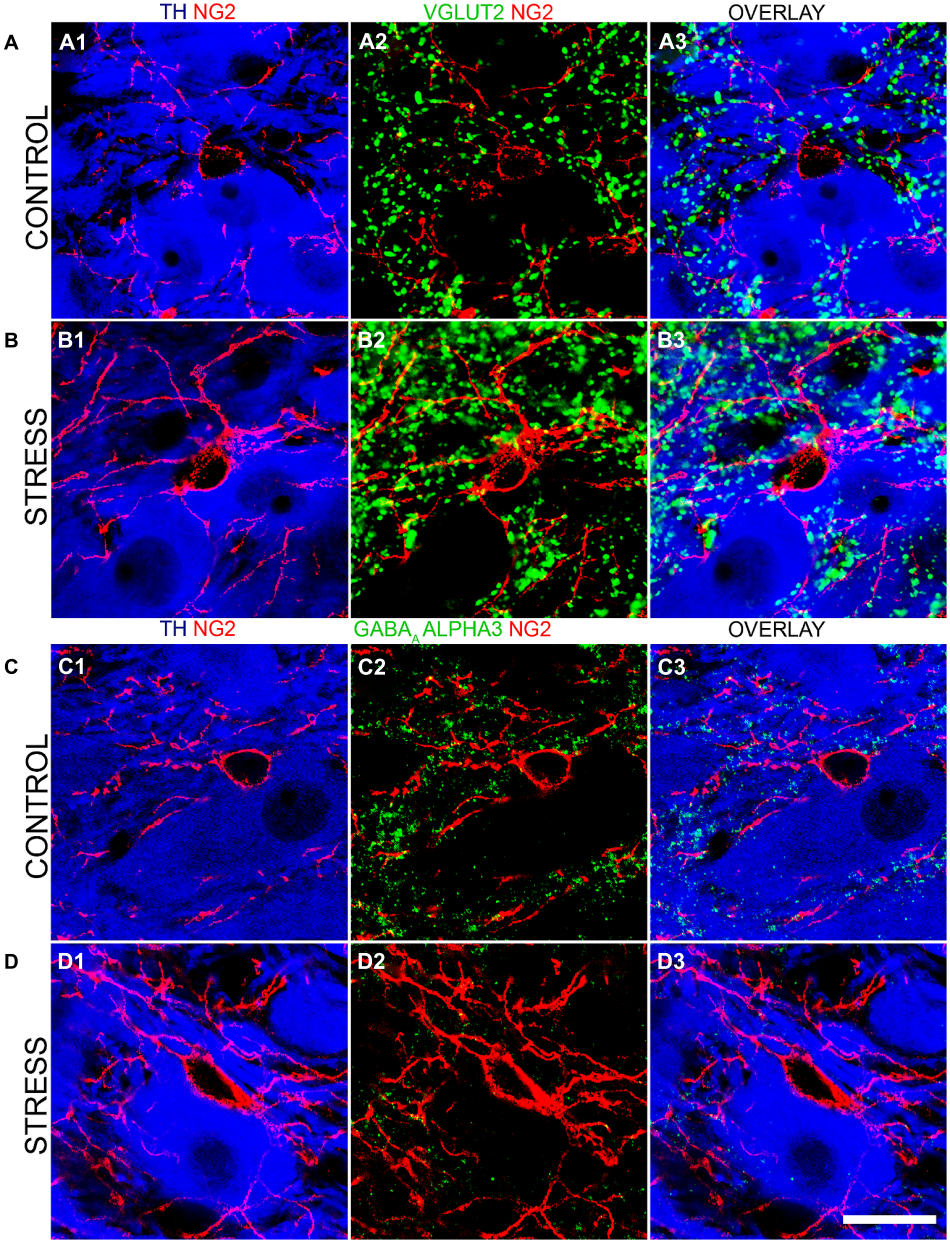
**Repeated restraint stress influences the intensity of immunoreactivity for excitatory and inhibitory synaptic marker proteins as well as their association with NG2 immunopositive profiles within the LC. (A1)** shows an NG2 immunopositive cell and its processes together with TH immunoreactive cell bodies and dendrites in tissue from a control animal. **(A2)** Shows immunoreactivity for the glutamatergic presynaptic protein, vesicular glutamate transporter 2 (VGLUT2) in relation to the NG2 cell in the corresponding field of view. **(A3)** is an overlay of **(A1)** and **(A2)**. **(B1)** Shows an NG2 immunopositive cell and its processes together with TH immunoreactive cell bodies and dendrites in tissue from a stress animal, reacted and imaged under conditions identical to those of control tissue. Note the significantly higher level of intensity for NG2 compared to control. **(B2)** Shows immunoreactivity for VGLUT2 in relation to an the NG2 cell in the corresponding field of view. Note the striking increase in the density of VGLUT2 immunopositive clusters and their strong association with NG2 immunoreactivity. **(B3)** is an overlay of **(B1)** and **(B2)**. **(C1)** shows an NG2 immunopositive cell and its processes together with TH immunoreactive cell bodies and dendrites in tissue from a control animal. **(C2)** Shows immunoreactivity for the GABA_A_R alpha3 subunit in relation to an the NG2 cell in the corresponding field of view. **(C3)** is an overlay of **(C1)** and **(C2)**. **(D1)** shows an NG2 immunopositive cell and its processes together with TH immunoreactive cell bodies and dendrites in tissue from a stress animal, reacted and imaged under conditions identical to those of control tissue. **(D2)** shows immunoreactivity for the GABA_A_R alpha3 subunit in relation to the NG2 cell in the corresponding field of view. Note the striking decrease in the density of GABA_A_R alpha3 subunit immunopositive clusters and their sparse association with NG2 immunoreactivity. **(D3)** is an overlay of **(D1)** and **(D2)**. Scale bar 20 μm.

To further investigate this stress-induced plasticity of NG2 cell profiles in the context of LC cellular networks, we evaluated whether there were any changes in the degrees of association between NG2 cell profiles and the synaptic marker proteins shown in **Figures 4** and **5**. There was a striking increase in the intensity of VGLUT2 immunoreactivity within the LC following the 10 days of the repeated restraint stress protocol (**Figures 8A,B**). Quantification of the total density of VGLUT2 immunoreactive clusters revealed a significant increase in tissue from stress animals compared to control (mean ± SEM; control, 647 ± 38 clusters per 10,000 μm^2^ versus stress, 1040 ± 32 clusters per 10,000 μm^2^; *P* = 0.0014, unpaired Student’s *t-*test, *N* = 5 animals in each group). Accordingly, there was also a higher proportion of VGLUT2 immunoreactive clusters which contacted NG2 immunoreactive profiles in tissue from stress animals compared to control (mean ± SEM; control, 100 ± 7 clusters per 10,000 μm^2^ versus stress, 529 ± 40; *P* = 0.004, unpaired Student’s *t-*test, *N* = 5 animals in each group).

In contrast to this stress-induced increase in the glutamatergic innervation of the LC, the level of immunoreactivity for the GABA_A_R alpha3 subunit, the principal GABA_A_R subtype within the LC and thus a key component of GABA_A_R-mediated inhibition of LC neuronal activity was significantly decreased (**Figures 8C,D**). Quantification of the total density of alpha3 subunit immunoreactive clusters revealed a significant decrease in tissue from stress animals compared to control (mean ± SEM; control, 130 ± 9 clusters per 10,000 μm^2^ versus stress, 82 ± 8 clusters per 10,000 μm^2^; *P* = 0.0136, unpaired Student’s *t-*test, *N* = 5 animals in each group). Accordingly, there was also a lower proportion of alpha3 subunit immunoreactive clusters which contacted NG2 immunoreactive profiles in tissue from stress animals compared to control (mean ± SEM; control, 18 ± 2 clusters per 10,000 μm^2^ versus stress, 8 ± 2; *P* = 0.0139, unpaired Student’s *t-*test, *N* = 5 animals in each group). No significant differences in the total density of gephyrin immunoreactivity (mean ± SEM; control, 125 ± 8 clusters per 10,000 μm^2^ versus stress, 132 ± 9 clusters per 10,000 μm^2^; *P* = 0.5565, unpaired Student’s *t-*test, *N* = 5 animals in each group) or the proportion of gephyrin immunoreactive clusters contacting NG2 immunoreactive profiles (mean ± SEM; control, 15 ± 1 clusters per 10,000 μm^2^ versus stress, 16 ± 2 clusters per 10,000 μm^2^; *P* = 0.6918, unpaired Student’s *t-*test, *N* = 5 animals in each group) were detectable in control and stress tissue.

## DISCUSSION

In the current study, we show that NG2 neuroglia cells are intimately associated with the principal neurons of the LC by virtue of their cell bodies being ensconced within somatic indentations of noradrenergic neurons and their processes contacting inhibitory synapses. Remarkably, repeated restraint stress, while not inducing an anxiogenic or hyper-locomotor behavioral phenotype, induced an increase in the density of NG2 cells selectively within the LC. It is currently unclear whether this stress-induced plasticity in the numbers of NG2 cells is a manifestation of newly generated NG2 cells within the LC or the migration of existing NG2 cells from neighboring brain regions. The use of various transgenic NG2 cell reporter mouse lines for cell-fate mapping (Rivers et al., 2008; Kang et al., 2010; Clarke et al., 2012) will prove invaluable in determining the origin of NG2 cells arising from emotive insults. Collectively, these data suggest that LC NG2 cell plasticity parallels with behavioral adaptations to emotive triggers raising the prospect that they cooperate with LC noradrenergic neurons in modulating the behavioral responses to aversive life events. If so, defining the precise functional roles of LC NG2 cells in either the adaptive or pathological stress pathways could provide unique avenues for either promoting resilience to stress, or possibly intervening therapeutically in terms of stress-induced mental illnesses.

Convergent lines of evidence point to NG2 cells serving as a source of OPCs which primarily give rise to oligodendrocytes (Kang et al., 2010; Young et al., 2013). The implication is that their main function in the CNS is that of myelination. It is thus intriguing why such cells are concentrated in a nucleus composed primarily of un-myelinated neurons, such as the LC. They could be involved in the myelination of LC afferents arising from diverse brain regions or be in the process of migrating to neighboring nuclei. However, the soma-somatic contacts of roughly 50% of NG2 cells with the un-myelinated noradrenergic neurons of the LC infer that this sub-population interact primarily with LC principal neurons. Further evidence for NG2 cells associating primarily with LC noradrenergic neurons is the precise positioning of NG2 cell processes with synaptic marker protein expression. The expression of the pan-neuronal marker exclusively within this cohort of NG2 cells located within somatic indentations leads us to conclude that this is a distinct population of NG2 cells which directly interacts with the output cells of the LC. This diversity of NG2 cell classes within the LC is in keeping with the rest of the brain where various populations have been defined based on their ability to generate electrical activity (Chittajallu et al., 2004; Karadottir et al., 2008; De Biase et al., 2010; Clarke et al., 2012), their molecular phenotypes (Gensert and Goldman, 2001; Mallon et al., 2002; Lin et al., 2009), their multi-potency (Dimou et al., 2008; Rivers et al., 2008; Guo et al., 2009, 2010) and their response to brain injury (Keirstead et al., 1998; Lytle et al., 2009; Kang et al., 2010). It is speculative whether this NeuN-immunopositive, sub-class of NG2 cells which is located within indentations of LC neurons represent those populations which show the rudimentary, action potential-like spiking activity described in other brain regions (Chittajallu et al., 2004; Karadottir et al., 2008) and thus are capable of reciprocal synaptic communication with LC noradrenergic neurons.

Structurally, such bi-directional synaptic communication seems unlikely since NG2 cells lack a defined axon by which they could relay any likely action potentials. However, the close soma-somatic contact with LC noradrenergic neurons might represent a yet to be described form of cellular communication. Based on the above data, the expectation is that NG2 cells receive excitatory input, from both glutamate and CRH-containing axons and modulate GABAergic synapses on LC noradrenergic neurons with the predicted result being the inhibition of LC neuronal activity and hence noradrenaline release. Simplistically, NG2 cell-mediated inhibitory modulation of LC neurons could serve as a negative feedback loop of the CRH-noradrenergic system preventing excessive neuronal excitation from any CRH released during times of stress (Valentino et al., 1983). If so, the stress-induced increase in the density of NG2 cells is likely to be a key mechanism by which the stress-induced LC output is tightly regulated allowing for the adoption of optimal behavioral strategies. Indeed, whilst this mild stress protocol predictably did not induce and anxiogenic phenotype when the animals were confronted with novelty in the form of the elevated plus maze, there was a significant degree of NG2 cell plasticity within the LC, a brain region expected to be integral to modulating such adaptive behavior (Valentino and Van Bockstaele, 2008). Importantly, since the proliferative potential of NG2 cells within the brain is finite, chronic periods of stress in adulthood might lead to the exhaustion of NG2 cell numbers within the LC. As a consequence, the absence of their homeostatic role during future stressful events might contribute to the dysregulation of the LC-noradrenergic system and the development of stress-induced mental illnesses.

In conclusion, the study provides the first demonstration of the dynamic expression patterns of NG2 neuroglia cells within the LC nucleus during development and behavioral states. The data suggest that NG2 cells are integral components of the LC cellular networks, are likely to be influenced by ongoing neuronal activity within this nucleus, and their precise functional contribution to coordinated network activity needs to be deciphered and contemplated when constructing models of LC function.

## Conflict of Interest Statement

The authors declare that the research was conducted in the absence of any commercial or financial relationships that could be construed as a potential conflict of interest.
